# Beyond Anticoagulation: Limitations of Oral Anticoagulants in Preventing Stroke Recurrence in Atrial Fibrillation

**DOI:** 10.3390/jcm13237309

**Published:** 2024-12-01

**Authors:** Jessica Seetge, Balázs Cséke, Zsófia Nozomi Karádi, Edit Bosnyák, László Szapáry

**Affiliations:** 1 Stroke Unit, Department of Neurology, University of Pécs, 7624 Pécs, Hungary; j.seetge@gmx.de (J.S.); karadi.zsofia@pte.hu (Z.N.K.); bosnyak.edit@pte.hu (E.B.); 2 Department of Emergency Medicine, University of Pécs, 7624 Pécs, Hungary; cseke.balazs@pte.hu

**Keywords:** real-world data, oral anticoagulants, recurrent stroke, cardioembolic, atrial fibrillation

## Abstract

**Background/Objectives**: Despite the widespread use of oral anticoagulants (OACs), acute ischemic stroke (AIS) remains a significant risk for patients with atrial fibrillation (AF). The real-world effectiveness of OACs in preventing recurrent strokes, particularly following an initial stroke of cardioembolic (CE) origin, continues to be a major challenge for clinicians managing AF patients. This study evaluated the efficacy of OACs in secondary stroke prevention and investigated the influence of anticoagulation type and quality on recurrence risk. **Methods**: We analyzed data from 128 AF patients in the prospective Transzlációs Idegtudományi Nemzeti Laboratórium (TINL) STROKE-registry, admitted with CE stroke between February 2023 and September 2024. Patients were categorized by anticoagulation status at admission (OAC-users, *n* = 89; anticoagulation-naïve, *n* = 39). Recurrence rates were assessed using logistic regression models, adjusted for age, sex, hypertension, diabetes, and pre-stroke disability. Subgroup analyses explored the effects of anticoagulation type and quality. **Results**: Recurrence rates were similar between the OAC-treated and anticoagulation-naïve patients after adjusting for confounders (19.10% vs. 17.95%, *p* = 0.870). Among the anticoagulated patients, neither anticoagulation type nor quality alone significantly influenced the recurrence risk. However, their interaction was statistically significant (*p* = 0.049), suggesting that the effectiveness of anticoagulation in preventing strokes is strongly affected by treatment quality. **Conclusions:** Although OACs are a cornerstone of stroke prevention in patients with AF, their efficacy in reducing recurrence depends on optimal management. These findings highlight that adequate anticoagulation, not just its use, is critical to minimize recurrence risk. To effectively prevent strokes in high-risk AF patients, future strategies must focus on standardized protocols, tailored monitoring, and individualized dosing regimens.

## 1. Introduction

Stroke is the second leading cause of death and a major contributor to long-term disability worldwide [1]. Approximately 87% of all strokes are ischemic, primarily caused by arterial occlusions, leading to cerebral ischemia and subsequent neuronal death [2]. A total of 27% of acute ischemic strokes (AISs) are cardioembolic (CE) in origin [3], with atrial fibrillation (AF) being a major risk factor, increasing the risk fivefold [4]. Furthermore, AF patients tend to experience more severe strokes and have poorer outcomes compared to those in sinus rhythm [5].

In patients with non-valvular atrial fibrillation (NVAF), direct oral anticoagulants (DOACs), such as factor Xa inhibitors and direct thrombin inhibitors, are recommended over vitamin K antagonists (VKAs) for stroke prevention [6]. Clinical trials have shown that DOACs reduce the risk of stroke by 64% and mortality by 26% compared to the placebo [7,8], and by 20% and 10%, respectively, compared to VKAs [9]. Despite these benefits, up to one-third of AF patients on anticoagulation therapy still suffer an AIS [10,11].

Secondary prevention strategies including antithrombotic agents, statins, and antihypertensives reduce the risk of secondary vascular events by 20% to 30% [12,13]. However, patients with AF who experience a stroke while on anticoagulation have an elevated risk of recurrence, with approximately 8.9% suffering an AIS annually [14]. CE strokes, in particular, carry the highest risk of recurrence, with one in four survivors likely to have another stroke [5,15].

Notably, there are currently no specific guidelines from the American Cardiology or Neurology Societies on preventing further ischemic events in patients who suffer a stroke despite being on anticoagulation [16,17]. As a result, the optimal prevention strategies for these high-risk patients remain uncertain [14,18,19]. This study aimed to evaluate the efficacy of OACs in secondary stroke prevention and explore how the type and quality of anticoagulation impact the risk of recurrence in patients with AF.

## 2. Materials and Methods

### 2.1. Study Design and Patient Population

We conducted a retrospective analysis using data from our prospective Transzlációs Idegtudományi Nemzeti Laboratórium (TINL) STROKE-registry. From February 2023 to September 2024, 1138 consecutive adult patients were admitted to the Department of Neurology, University of Pécs. Of these, 1010 patients were excluded for the following reasons: acute hemorrhagic stroke (AHS) (*n* = 66), transient ischemic attack (TIA) (*n* = 152), non-cardioembolic stroke (*n* = 604), or no prior diagnosis of atrial fibrillation (*n* = 188). Ultimately, 128 patients met the inclusion criteria and were enrolled in the study.

The final cohort was divided into two groups: patients receiving oral anticoagulation (OAC group, *n* = 89) and those who were anticoagulation-naïve (Non-OAC group, *n* = 39). The OAC group was further categorized according to the type: DOAC (*n* = 66) or VKA (*n* = 23), and quality of anticoagulation: under-anticoagulated (*n* = 34), appropriately anticoagulated (*n* = 48), or over-anticoagulated (*n* = 7).

**Figure 1:**
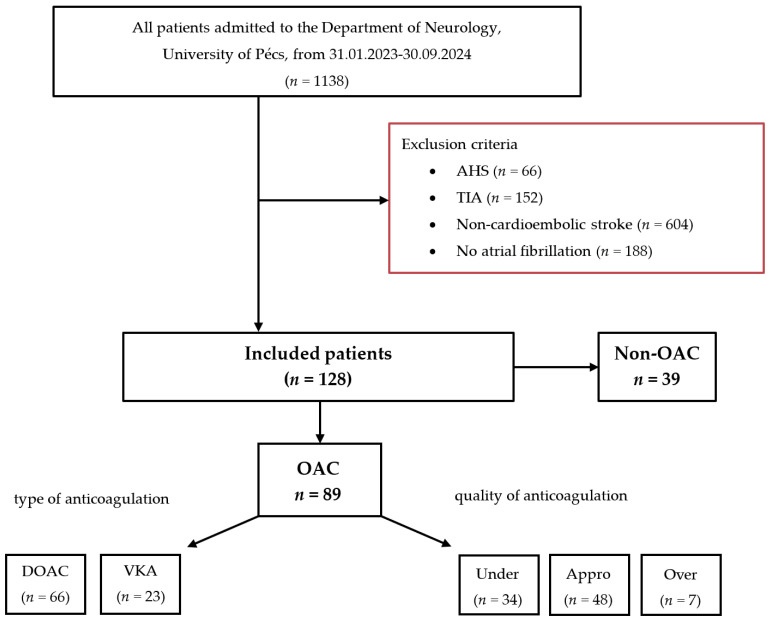
Flowchart of patients included in the study. Abbreviations: AHS = acute hemorrhagic stroke, TIA = transient ischemic attack, DOAC = direct oral anticoagulant, VKA = vitamin K antagonist, Under = under-anticoagulated, Appro = appropriately anticoagulated, Over = over-anticoagulated.

Inclusion criteria for the study were: (1) AIS of CE origin (referred to as index stroke), diagnosed through a combination of typical clinical presentation (sudden onset of neurological deficits, commonly involving the cerebral cortex), neuroimaging findings (involvement of cortical territory lesions), and evidence of a high-risk cardiac source; (2) an electrocardiogram (ECG)-confirmed diagnosis of NVAF established before the index stroke; and (3) the availability of detailed information on anticoagulation therapy before the index stroke. A flowchart summarizing the patient exclusion and inclusion criteria is provided in Figure 1.

### 2.2. Data Collection and Measurements

This registry includes comprehensive demographic and clinical data such as age, sex, and medical history (e.g., current smoking, alcohol use, hypertension, and diabetes mellitus). Details on anticoagulant treatment (no treatment, VKA, or DOAC including the specific type), along with weight, creatinine, and glomerular filtration rate (GFR) at admission (for patients receiving DOAC therapy) or international normalized ratio (INR) at the index stroke (for patients receiving VKA therapy) were also recorded. Pre-stroke disability was assessed using the premorbidity modified Rankin Scale (pre-mRS), and stroke severity at admission was evaluated using the National Institutes of Health Stroke Scale (NIHSS). Additional data points included the functional and safety outcomes of the index stroke and history of CE stroke (including the interval between strokes and whether AF was known before the previous CE stroke).

DOAC therapy was defined as treatment with one of the following drugs and dosages: apixaban (2.5 mg or 5 mg twice daily), edoxaban (30 mg or 60 mg once daily), rivaroxaban (15 mg or 20 mg once daily), or dabigatran (110 mg or 150 mg twice daily). VKA therapy was defined as treatment with warfarin or acenocoumarol.

Anticoagulation quality was categorized as follows: under-anticoagulation, defined as an inappropriate dose reduction in DOAC patients or an admission-INR below 2.0 for VKA patients. Appropriate anticoagulation: patients who met the dosing requirements in accordance with the current guidelines. Over-anticoagulation was defined as inappropriate dosing without a dose reduction in DOAC patients or an admission-INR above 3.0 for VKA patients.

### 2.3. Follow-Up and Outcome Measures

Follow-up was conducted for at least three months post-index stroke to assess the following: functional outcome at 90 days using the modified Rankin Scale (mRS), all-cause mortality during the follow-up period, and intracerebral hemorrhage (ICH), defined as new neurological symptoms associated with intracerebral hemorrhage confirmed by computer tomography (CT).

The primary endpoint was to assess the relationship between OAC use and the risk of recurrent CE stroke using prior CE stroke as a proxy measure. Recurrent stroke was defined as new symptomatic neurological deterioration, not due to non-ischemic causes, with imaging evidence confirming new brain infarction.

Additionally, a subgroup analysis was performed on the anticoagulated cohort to examine the impact of anticoagulation type and quality on recurrence risk. For patients on DOACs, dosages were evaluated based on weight, creatinine levels, and GFR at admission. For those on VKAs, anticoagulation quality was assessed using INR measurements at the time of admission.

### 2.4. Statistical Analyses

Data analysis was performed using the Statistical Product and Service Solutions (SPSS) software (version 23) and Python (version 3.13.0). The normality of independent continuous variables was assessed using both descriptive and analytical criteria. Baseline characteristics were summarized through descriptive statistics. Continuous variables were expressed as mean ± standard deviation (SD) or median with interquartile range (IQR) and compared using the Mann–Whitney U test. Categorical variables were presented as counts or percentages and analyzed using the *χ*^2^ test, Fisher’s exact test, or one-way analysis of variance (ANOVA) for subgroup comparisons.

Differences between groups in demographic or clinical characteristics were evaluated using Fisher’s exact test for binary data. The rate of stroke recurrence was evaluated using logistic regression, adjusted for potential confounders (e.g., age, sex, hypertension, diabetes, and pre-stroke disability). Additionally, we used two-way ANOVA, ridge regression with bootstrapping, and a gradient boosting model to assess both linear and nonlinear predictors in patients receiving anticoagulation.

All statistical tests were two-tailed, with a *p*-value of <0.05 considered statistically significant. Odds ratios (OR) with 95% confidence intervals (CI) were reported.

## 3. Results

### 3.1. Demographic and Clinical Characteristics

A total of 128 AF patients with AIS were retrospectively reviewed, of whom 89 (69.5%) were on anticoagulation therapy (OAC group: 43.8% male, median age 80 years [IQR: 46–95]). The remaining 39 patients (30.5%) were not on anticoagulation therapy (Non-OAC group: 41.0% male, median age 81 years [IQR: 58–93]).

Clinical characteristics included a pre-mRS score of 0 (IQR, 0–4) for the OAC group and 0 (IQR: 0–5) for the Non-OAC group. The median NIHSS score was 6 (IQR: 0–36) in the OAC group and 7 (IQR: 2–21) in the Non-OAC group. Detailed demographic and clinical characteristics are summarized in Table 1.

### 3.2. Functional and Safety Outcomes

No significant differences were observed between the OAC and Non-OAC groups regarding functional outcomes or mortality at 90 days. Both groups had a median 90-day mRS of 5 (IQR: 0–6, *p* = 0.622). Mortality at 90 days was similar between groups (33.7% for OAC vs. 30.8% for Non-OAC, *p* = 0.682). Although the incidence of ICH was higher in the OAC group (6.7% vs. 0.0%), this difference was not statistically significant (*p* = 0.465).

### 3.3. Previous History of CE Stroke

**Figure 2:**
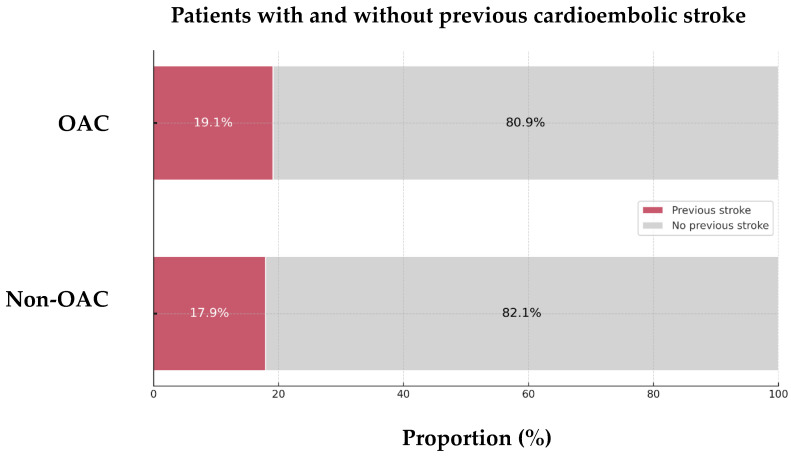
Proportion of previous cardioembolic strokes in the 128 AIS-AF patients. Abbreviations: AIS = acute ischemic stroke, AF = atrial fibrillation, OAC = oral anticoagulant.

Of the 128 patients, 24 (18.75%) had a history of prior CE stroke. Among these, 19.1% were on anticoagulation therapy, while 17.9% were anticoagulation-naïve before the index stroke (*p* = 0.870, Figure 2). NVAF was present in roughly half of the patients before the previous CE stroke, regardless of prior anticoagulation status (41.2% in those on anticoagulation vs. 42.9% in those without).

Anticoagulated patients who experienced a recurrent stroke had a longer median interval between strokes compared to those who were not anticoagulated (6 years vs. 3 years, *p* = 0.672), suggesting a potential protective effect of anticoagulation therapy (Table 2).

### 3.4. Factors Influencing Recurrence Risk

**Figure 3:**
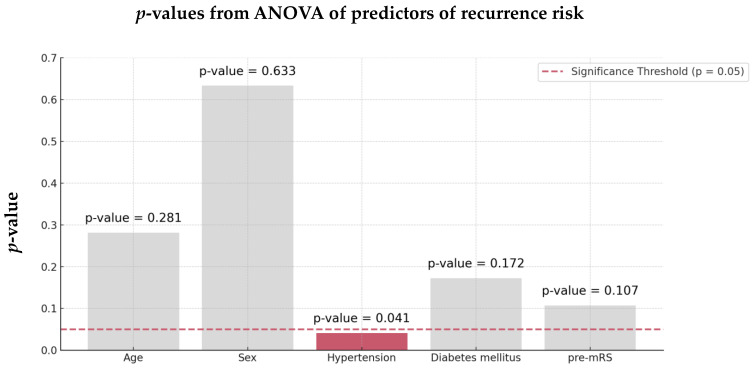
ANOVA of the predictors of recurrence risk of the 128 AIS-AF patients. Abbreviations: ANOVA = analysis of variance, AIS = acute ischemic stroke, AF = atrial fibrillation.

ANOVA identified hypertension as the most significant factor associated with recurrent stroke in both groups (*p* = 0.041, Figure 3).

### 3.5. Subgroup Analysis of Anticoagulated Patients

**Figure 4:**
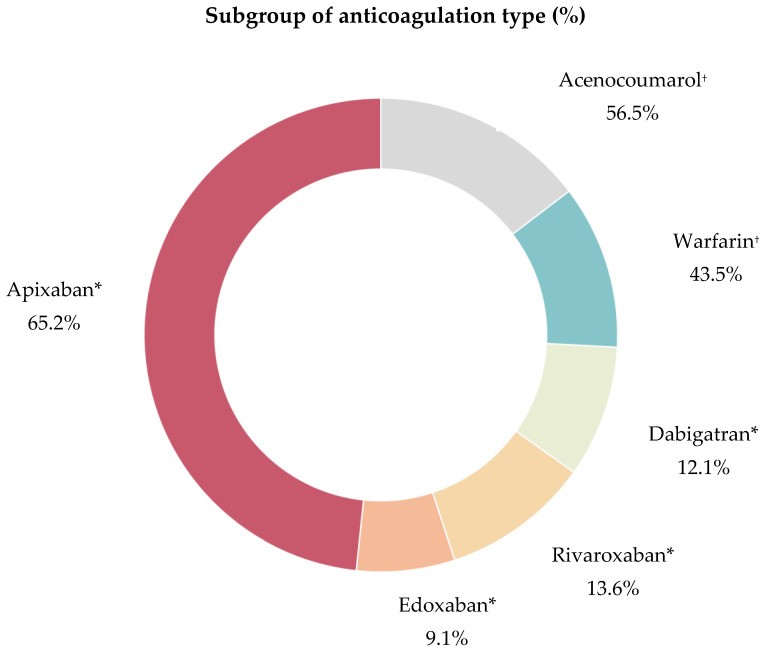
Subgroup of anticoagulation type of the 89 AIS-AF patients. Abbreviations: AIS = acute ischemic stroke, AF = atrial fibrillation, * add up to 100%, ^†^ add up to 100%.

In our subgroup analysis of 89 OAC patients, the majority (74.2%) were on DOACs, with apixaban being the most commonly prescribed (65.2%). The remaining 25.8% were on VKAs, mainly acenocoumarol (56.5%) (Figure 4).

At admission, 53.9% of all anticoagulated patients were adequately anticoagulated, while 38.2% were under-anticoagulated, and 7.9% were over-anticoagulated (Table 3).

**Figure 5:**
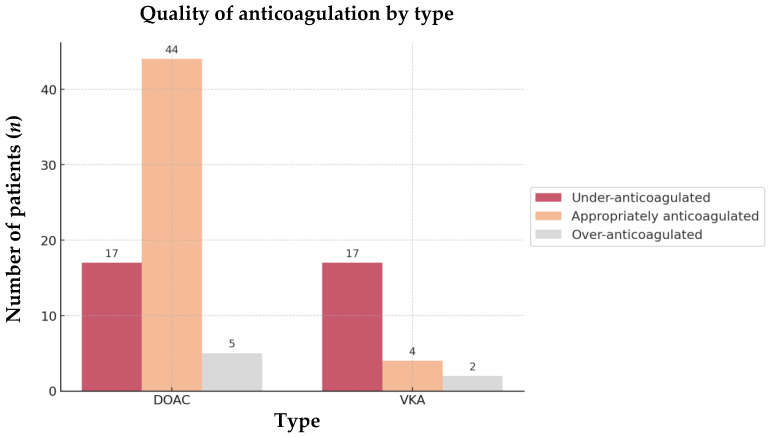
Quality of anticoagulation by type of the 89 OAC AIS-AF patients. Abbreviations: OAC = oral anticoagulant, AIS = acute ischemic stroke, AF = atrial fibrillation, DOAC = direct oral anticoagulant, VKA = vitamin K antagonist.

A total of 66.7% of patients taking DOACs were appropriately anticoagulated compared to only 17.4% in the VKA group (OR = 9.5; 95% CI, 0.07–0.37; *p* < 0.001). Furthermore, under-anticoagulation was significantly less common in the DOAC group (25.8%) than in the VKA group (73.9%) (OR = 0.12; 95% CI, 0.04–0.36; *p* < 0.001) (Figure 5).

**Figure 6:**
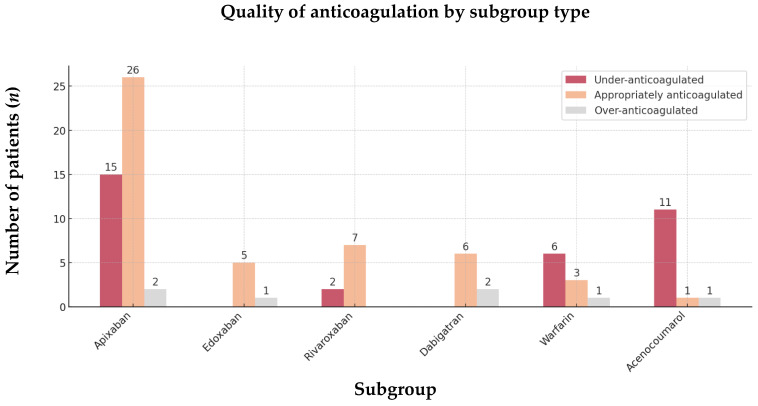
Quality of anticoagulation by subgroup type of the 89 OAC-AIS-AF patients. Abbreviations: OAC = oral anticoagulant, AIS = acute ischemic stroke, AF = atrial fibrillation.

The comparison across different subgroups of VKA and DOAC highlights the same trend (Figure 6).

### 3.6. Type of Anticoagulant

Stratification by anticoagulation type revealed no significant differences in the baseline characteristics between patients taking DOACs and those taking VKAs (Table 4).

**Figure 7:**
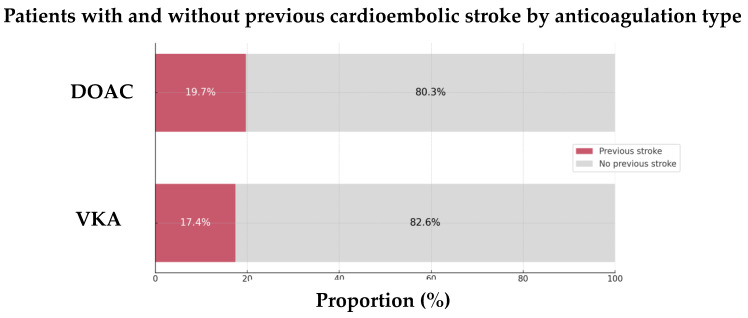
Proportion of previous cardioembolic strokes by anticoagulation type in the 89 AIS-AF patients. Abbreviations: AIS = acute ischemic stroke, AF = atrial fibrillation, DOAC = direct oral anticoagulant, VKA = vitamin K antagonist.

**Figure 8:**
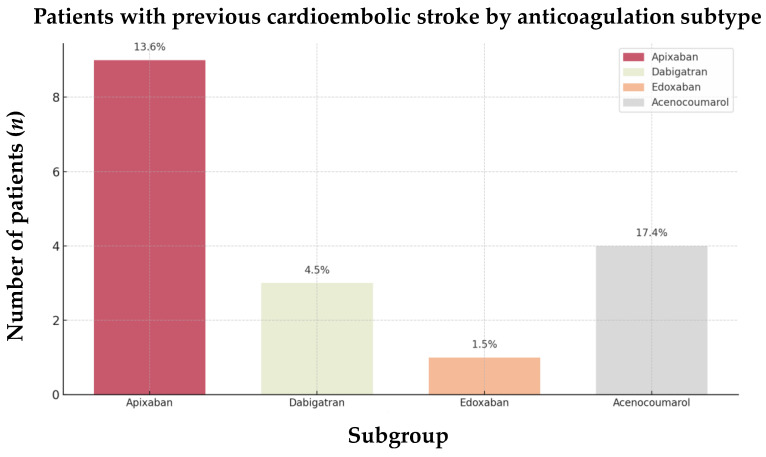
Proportion of previous cardioembolic strokes by anticoagulation subtype in the 17 AIS-AF patients. Abbreviations: AIS = acute ischemic stroke, AF = atrial fibrillation.

These findings also hold for comparisons of functional and safety outcomes and the prevalence of previous CE strokes between the groups (Figure 7 and Figure 8). Among the patients with recurrent strokes, those on VKAs tended to have a shorter interval between strokes (5 years vs. 6 years, *p* = 0.741) and were more likely to have been diagnosed with AF prior to their previous CE stroke (13.0% vs. 6.1%, Table 5).

### 3.7. Quality of Anticoagulant

Stratification by anticoagulation quality revealed no significant differences in the baseline characteristics between the under-, appropriately, and over-anticoagulated patients (Table 6).

**Figure 9:**
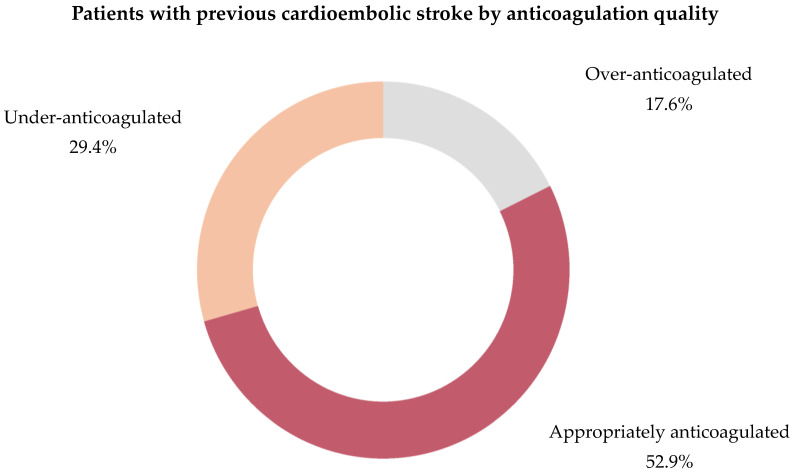
Proportion of previous cardioembolic strokes by anticoagulation quality in the 17 AIS-AF patients. Abbreviations: AIS = acute ischemic stroke, AF = atrial fibrillation.

These findings were consistent when comparing the functional and safety outcomes and the prevalence of previous CE strokes across the three groups (Figure 9).

Under-anticoagulated patients were more likely to have been diagnosed with AF prior to their previous CE stroke (80.0%). Given that a majority of patients with VKA were under-anticoagulated (73.9%), it is likely that inadequate anticoagulation may have contributed to their prior strokes (Table 7).

Age, hypertension, diabetes mellitus, and pre-mRS were significant predictors of recurrent cardioembolic stroke among the anticoagulated patients. Each additional year of age was associated with a 30.2% increase in the odds of recurrence (mean coefficient: 0.2644, 95% CI: 0.0219–0.4131). Similarly, patients with diabetes had a notably higher recurrence risk (mean coefficient: 0.1417, 95% CI: 0.0335–0.2599).

Conversely, anticoagulated patients with hypertension (mean = −0.1404; 95% CI, −0.2562, −0.0207) and higher pre-mRS scores (mean = −0.1866; 95% CI, −0.2893, −0.0795) appeared to have a lower risk of recurrence, which might initially seem counterintuitive. This finding can be explained by the possibility that patients with poorly controlled hypertension or higher pre-mRS scores may not have survived long enough to experience a second stroke within the timeframe of our analysis. Furthermore, those who survived likely benefited from more aggressive hypertension management post-stroke, thereby reducing their recurrence risk.

To capture more complex patterns, we applied a gradient boosting model paired with Shapley Additive exPlanation (SHAP) analysis, revealing that both hypertension and high pre-mRS scores were in fact linked to an increased risk of recurrence.

**Figure 10:**
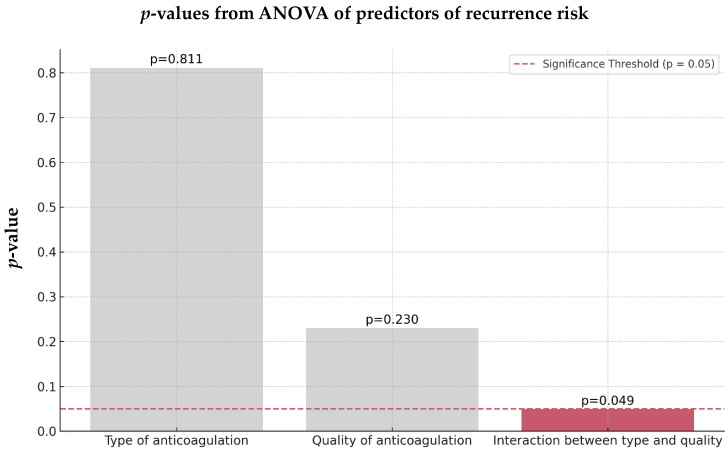
ANOVA predictors of the recurrence risk of the 89 OAC AIS-AF patients. Abbreviations: OAC = oral anticoagulant, AIS = acute ischemic stroke, AF = atrial fibrillation.

Interestingly, neither the type nor the quality of anticoagulation alone significantly affected the risk of recurrence in the OAC group. However, there was a significant interaction between anticoagulation type and quality (*p* = 0.049), indicating that the effectiveness of OACs in reducing a recurrent stroke is strongly influenced by maintaining high-quality treatment (Figure 10).

**Figure 11:**
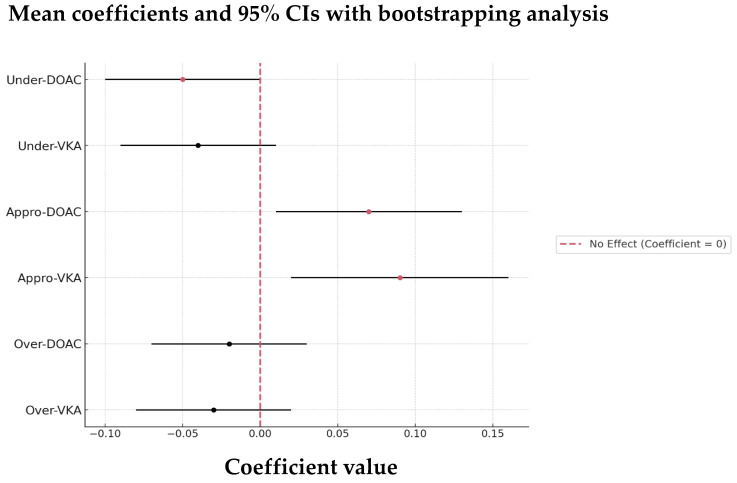
Ridge regression with bootstrapping analysis of the predictors of recurrence risk of the 89 OAC AIS-AF patients. Abbreviations: OAC = oral anticoagulant, AIS = acute ischemic stroke, AF = atrial fibrillation, DOAC = direct oral anticoagulant, VKA = vitamin K antagonist, Under = under-anticoagulated, Appro = appropriately anticoagulated, Over = over-anticoagulated.

Looking closer at this interaction, we observed a trend showing that appropriately anticoagulated patients on either DOAC or VKA therapy may have a reduced risk of recurrence, suggesting that patients receiving optimal anticoagulation are less likely to have another stroke compared to those who are under- or over-anticoagulated (Figure 11).

## 4. Discussion

Our study found no significant difference in the recurrence rates between the OAC-treated and anticoagulation-naïve patients after adjusting for confounding factors. Neither the type nor the quality of anticoagulation alone showed a significant impact on recurrence risk. However, the interaction between type and quality was significant. Achieving optimal anticoagulation, whether with DOACs or VKAs, was associated with a lower risk of recurrence, highlighting the importance of maintaining high-quality anticoagulation in AF patients.

The absence of a significant impact of anticoagulant type or quality alone can be explained by multicollinearity (when analyzed independently, both factors may lose meaningful interactions that are critical for understanding their actual effects) or power and sample size (evaluating anticoagulation type and quality separately might have resulted in insufficient statistical power to detect significant effects). In contrast, the combined interaction between both was powerful enough to demonstrate a significant influence on recurrence risk.

### 4.1. Mechanisms of Stroke Despite OACs

Despite the use of OACs, a residual risk of stroke persists, which could be due to several factors including: competing stroke mechanisms unrelated to AF (such as large-artery disease and small-vessel disease), insufficient anticoagulation (which may result from non-compliance, low anticoagulant activity at admission, inappropriate low-dose DOAC usage according to product labeling, or temporary withdrawal of therapy), and other CE mechanisms distinct from AF [20,21,22].

#### 4.1.1. Non-AF-Related Mechanisms

Approximately 30% of recurrent strokes are not related to AF [17]. Within this subgroup, the most common competing mechanism is large artery atherosclerosis (60.6%), followed by small vessel disease (26.3%) [18]. For many of these stroke mechanisms, anticoagulation offers no advantage over aspirin. For example, the Warfarin-Aspirin Recurrent Stroke Study (WARSS) demonstrated no benefit of warfarin compared to aspirin in patients with non-CE stroke [23]. Similarly, the Warfarin-Aspirin Symptomatic Intracranial Disease (WASID) trial found that warfarin was not superior to aspirin for secondary stroke prevention in patients with intracranial atherosclerosis [24].

#### 4.1.2. Insufficient Anticoagulation Therapy

Despite having significant embolic risk factors, only half of the patients with NVAF currently use Food and Drug Administration (FDA)-approved methods for stroke prevention [25]. Likewise, adherence to anticoagulation therapy remains a challenge, with many patients discontinuing treatment or receiving inadequate doses [26]. In a retrospective cohort analysis of 16,075 AF patients who initiated either warfarin or a DOAC, it was found that more than one in eight patients discontinued therapy within the first year [27]. Additionally, a 2016 study reported declining persistence rates over a 12 month-period, with warfarin showing a persistence rate of 63.6%, while DOACs had a slightly higher persistence rate of 79.2% among newly diagnosed AF patients [28].

Recent evidence underscores the significant risks associated with discontinuing OACs following an ischemic stroke. A Danish nationwide cohort study involving 8119 patients 50 years and older (54.1% male, mean age 78.4 years) showed that approximately 4.3% experienced a recurrent stroke within one year after discharge from their initial stroke. Importantly, those who discontinued anticoagulation were found to be more than twice as likely to experience another stroke during a mean follow-up period of 2.9 years compared to those who continued therapy [29]. These findings highlight the critical need for strategies to improve adherence and reduce the discontinuation rates.

Among the patients enrolled in the warfarin arms of the three most widely used DOAC trials (ROCKET-AF, RE-LY, and ARISTOTLE), the average time within the therapeutic range was notably low, ranging from 55% to 62% [30,31,32]. Underdosing of DOACs also presents a significant concern. Many patients are prescribed reduced doses, often with the aim of mitigating bleeding risks under the principle of “do no harm”. Similarly, in our study, under-anticoagulation was a prevalent issue, affecting a significant portion of both VKA and DOAC patients. The FDA has previously issued warnings against the off label underdosing of anticoagulants, highlighting the associated risks of compromised safety and efficacy [33,34].

#### 4.1.3. CE Stroke Despite Adequate Anticoagulation Therapy

CE stroke in AF patients despite optimal anticoagulation therapy represents a significant challenge in clinical practice, mainly because the underlying mechanisms are not yet fully understood. Evidence suggests that advanced atrial disease including atrial enlargement and increased AF burden, along with high CHA_2_DS_2_-VASc scores, may contribute to ischemic strokes in these patients. This phenomenon indicates that CE risk may persist even under adequate anticoagulation, pointing to a gap in the current therapeutic strategies for stroke prevention in high-risk AF patients [35].

### 4.2. Embolic Stroke of Undetermined Source (ESUS)

Embolic stroke of undetermined source (ESUS) refers to a subset of ischemic strokes where an embolic origin is suspected, but no definitive source can be identified after ruling out other possible stroke etiologies. Two major randomized clinical trials “Randomized, Double-Blind, Evaluation in Secondary Stroke Prevention Comparing the Efficacy and Safety of Dabigatran Etexilate Versus Acetylsalicylic Acid in Patients With Embolic Stroke of Undetermined Source” (RE-SPECT ESUS) and “New Approach Rivaroxaban Inhibition of Factor Xa in a Global Trial Versus Aspirin to Prevent Embolism in Embolic Stroke of Undetermined Source” (NAVIGATE ESUS) aimed to determine the best treatment approach for ESUS. Both trials concluded that neither dabigatran nor rivaroxaban offered a significant advantage over aspirin in preventing recurrent strokes in ESUS patients [36,37]. Likewise, ATTICUS and ARCADIA demonstrated no benefit of apixaban over aspirin in preventing recurrent AIS among patients with ESUS [38,39]. These results challenged the expectation that empiric anticoagulation could significantly lower the risk of stroke recurrence compared to antiplatelet therapy.

Two additional trials examined the effectiveness of anticoagulation in patients with atrial high-rate episodes (AHRE), providing insights into the limitations of anticoagulation for secondary stroke prevention: “Non-vitamin K Antagonist Oral Anticoagulants in Patients With Atrial High Rate Episodes—Atrial Fibrillation Network 6” (NOAH-AFNET 6) and “Apixaban for the Reduction of Thrombo-Embolism in Patients with Device-Detected Subclinical Atrial Fibrillation” (ARTESiA). The NOAH-AFNET 6 trial was terminated prematurely due to increased major bleeding in the edoxaban group and futility assessment to achieve efficacy endpoints [40]. The second trial showed a significant reduction in stroke or systemic embolism in apixaban-assigned patients, but this benefit was counterbalanced by a significantly increased risk of major bleeding. The AIS rate was 0.64% per year in the apixaban group compared to 1.02% per year in the aspirin group, which, while lower, still represented a modest benefit relative to the risk of bleeding [41].

### 4.3. Risk Factors

In patients with AF, several risk factors are associated with an increased risk of ischemic stroke including older age, hypertension, diabetes, heart failure, previous stroke or TIA, vascular disease, renal dysfunction, low body mass index (BMI), and female sex [42]. Likewise, in our study, older age, hypertension, and diabetes mellitus emerged as independent factors associated with recurrent stroke in anticoagulated patients.

The high residual stroke risk in these patients must be quantified for targeted treatment. The currently used CHA_2_DS_2_-VASc score may not be adequate to assess the residual stroke risk as it weighs the history of previous strokes with a score of only 2. Furthermore, the CHA_2_DS_2_-VASc score does not account for the added risk associated with recurrent strokes in patients already receiving anticoagulation therapy.

#### 4.3.1. Hypertension

Hypertension is a well-established, independent risk factor for stroke recurrence. A meta-analysis involving 13,944 stroke survivors found that hypertension increased the odds of recurrent stroke by 67% (95% CI, 45–92%) over a follow-up period ranging from one to five years [43]. Additionally, data from the “Prevention Regimen for Effectively avoiding Second Strokes” (PROFESS) trial demonstrated that systolic blood pressure (SBP) variability was linked to an increased risk of recurrent stroke, where each 10-point increment in SBP variability was associated with a 15% higher hazard (95% CI, 2–32%) of recurrence [44]. In our study, hypertension was identified as the main risk factor for stroke recurrence in both anticoagulated and anticoagulation-naïve patients.

#### 4.3.2. Diabetes Mellitus

Diabetes mellitus is also a significant predictor of stroke recurrence. A meta-analysis of 27 studies involving 274,631 patients with prior ischemic stroke found that diabetes was associated with a 50% increase in the risk of stroke recurrence (pooled HR, 1.50; 95% CI, 1.36–1.65) [45].

### 4.4. Alternative Strategies and Future Directions

#### 4.4.1. Switching Anticoagulants

Observational studies have shown no benefit in reducing stroke recurrence by switching anticoagulation therapies or adding antiplatelet agents. Among the 2337 patients with NVAF, those who switched from one DOAC to another or converted to warfarin had higher rates of recurrent ischemic stroke (12.8% and 12.6%, respectively) compared to those who continued their initial DOAC (8.7%). Moreover, adding antiplatelet agents offered no additional benefit and was associated with an increased risk of bleeding complications [18,19].

#### 4.4.2. Percutaneous Left Atrial Appendage Closure (LAAC)

As of the time of this article, the only FDA-approved alternatives for stroke prophylaxis that eliminate the need for lifelong anticoagulation in patients with NVAF are LAAC devices, specifically WATCHMAN FLX and Amplatzer Amulet [46]. Two large-scale multinational randomized control trials (RCTs), the CHAMPION-AF trial for WATCHMAN FLX and the CATALYST trial for Amplatzer Amulet, are currently enrolling patients with NVAF to compare their efficacy against DOACs.

Moreover, several ongoing RCTs are investigating the potential benefits of combining endovascular LAAC with DOAC therapy including the “Evaluation of Percutaneous Left Atrial Appendage Occlusion in Stroke Prevention on Top of Direct Oral Anticoagulant Therapy” (ELPASE) trial.

A real-world comparative study showed that LAAC reduced the risk of stroke and long-term bleeding by approximately 35% compared to long-term oral anticoagulant use in patients with NVAF [47]. Furthermore, the “Left Atrial Appendage Occlusion Study III” (LAAOS III) RCT demonstrated that closing the left atrial appendage (LAA) during cardiac surgery for anticoagulated patients with AF led to a 33% reduction in the risk of AIS after the procedure [48]. In a separate study involving 115 patients who underwent endovascular LAAC after experiencing an AIS despite being on OAC, the observed annual rate of ischemic events was 2.6% after the procedure [49].

#### 4.4.3. Factor XIa Inhibitors

Following promising safety outcomes, phase 3 trials have been launched to evaluate asundexian (“Oral Factor XIa Inhibitor Asundexian in Patients with Atrial Fibrillation” [OCEANIC-AF]) and milvexian (“LIBREXIA-Atrial Fibrillation: Evaluation of Milvexian in Stroke Prevention for Atrial Fibrillation Patients” [LIBREXIA-AF]) for stroke prevention in patients with atrial fibrillation. Unfortunately, the OCEANIC-AF trial was prematurely stopped after an interim analysis revealed no significant efficacy.

In parallel, a phase 2b study (AZALEA-TIMI 71) comparing abelacimab to rivaroxaban showed a 67% reduction in the primary endpoint of major or clinically relevant non-major bleeding. Abelacimab is currently being studied in patients with atrial fibrillation who are not eligible for anticoagulation, however, individuals with a history of stroke are excluded from this trial.

### 4.5. Limitations

This study has several limitations that should be considered when interpreting the results. Firstly, the relatively small sample size from a single center and the non-randomized study design may have introduced potential selection bias, which may limit the generalizability of our findings. Although we used multivariable analyses to adjust for confounding variables, it was not possible to fully eliminate biases inherent to an observational design.

Additionally, the influence of unmeasured confounders cannot be ruled out. The absence of specific details on cardiac comorbidities limits our ability to fully characterize the cohort, making it challenging to compare our findings to those reported in the existing literature. Furthermore, important parameters associated with the risk of ischemic stroke in NVAF patients, such as the type of atrial fibrillation (sustained or paroxysmal), AF duration, untreated obstructive sleep apnea, and levels of natriuretic peptides or troponins, were not captured, which may have impacted the observed results [50,51,52].

Moreover, the study did not evaluate other pharmacological treatments beyond anticoagulation, such as antiplatelet therapy, and no data on plasma DOAC levels were available for our patients. This limits our assessment of the full efficacy of treatment, particularly concerning optimal anticoagulant dosing.

It should also be acknowledged that the effectiveness and safety of anticoagulation therapy may differ for patients who have experienced a more recent stroke and are at a heightened risk of recurrence [53].

While the retrospective design does limit our ability to establish causality or fully capture temporal changes in risk, our study still provides meaningful insights through its real-world perspective. This approach offers a valuable preliminary understanding of recurrent stroke risk among patients treated with OACs, capturing the practical challenges of anticoagulation therapy that are often missed in controlled settings. These findings are particularly important for developing strategies aimed at reducing recurrent strokes in AF patients receiving OAC therapy.

Notably, the observed lack of significant differences in the recurrence rates between anticoagulated and non-anticoagulated groups points to the underlying complexity of stroke recurrence mechanisms, suggesting that anticoagulation alone may not fully address all risk factors. This highlights important areas for future research to further explore the multifactorial nature of stroke recurrence.

## 5. Conclusions

Adequate anticoagulation, not just its use, is critical for minimizing the residual risk of stroke, especially in high-risk patients with AF who have suffered a previous CE stroke. Clinicians should focus on optimizing anticoagulation therapy, as inadequate treatment quality is associated with an increased recurrence risk. Future strategies should concentrate on individualized patient management, regular monitoring, and optimal dosing to effectively prevent further strokes.

Beyond anticoagulation, managing modifiable risk factors, such as hypertension and diabetes, is crucial. Emerging therapies such as LAAC have shown promise as alternative prevention strategies, offering outcomes comparable to DOACs. However, persistent recurrence rates suggest the need for combined approaches, such as integrating LAAC with anticoagulation, to further improve patient outcomes. Ultimately, this comprehensive approach may best serve high-risk AF patients and significantly reduce their risk of recurrent stroke.

## Figures and Tables

**Table 1:** Demographic and clinical characteristics of 128 OAC and Non-OAC AIS-AF patients.

	OAC (*n* = 89)	Non-OAC (*n* = 39)	*p*-Value/OR [95% CI]
Demographic characteristics			
Age, years, median (IQR)	80 (46–95)	81 (58–93)	*p* = 0.904
Sex, male, *n* (%)	39 (43.8%)	16 (41.0%)	OR = 1.12 [0.52–2.40], *p* = 0.780
Medical history, *n* (%)			
Current smoking	6 (6.7%)	3 (7.7%)	OR = 0.87 [0.21–3.66], *p* = 0.850
Alcohol	39 (43.8%)	14 (35.9%)	OR = 1.39 [0.64–3.03], *p* = 0.399
Hypertension	83 (93.3%)	37 (94.9%)	OR = 0.81 [0.18–3.62], *p* = 0.677
Diabetes mellitus	37 (41.6%)	19 (48.7%)	OR = 0.86 [0.39–1.90], *p* = 0.717
pre-mRS score, median (IQR)	0 (0–4)	0 (0–5)	*p* = 0.720
pre-mRS score, *n* (%)			
0	51 (57.3%)	22 (56.4%)	OR = 0.86 [0.43–1.73], *p* = 0.677
1	12 (13.5%)	8 (20.5%)	OR = 0.65 [0.24–1.74], *p* = 0.394
2	11 (12.4%)	0 (0.0%)	*p* = 0.008
>2	15 (16.9%)	9 (23.1%)	OR = 0.68 [0.27–1.71], *p* = 0.323
NIHSS score at admission, median (IQR)	6 (0–36)	7 (2–21)	*p =* 0.873

Abbreviations: OAC = oral anticoagulant, AIS = acute ischemic stroke, AF = atrial fibrillation, OR = (common) odds ratio, IQR = interquartile range, pre-mRS = premorbidity modified Rankin Scale, NIHSS = National Institute of Health Stroke Scale.

**Table 2:** Outcomes of the 128 OAC and Non-OAC AIS-AF patients.

	OAC (*n* = 89)	Non-OAC (*n* = 39)	*p*-Value/OR [95% CI]
Functional and safety outcome			
90-day mRS, median (IQR)	5 (0–6) *n* = 82	5 (0–6) *n* = 36	*p* = 0.622
Mortality at 90 days, *n* (%)	30 (33.7%)	12 (30.8%)	OR = 1.14 [0.51–2.57], *p* = 0.682
ICH, *n* (%)	6 (6.7%)	0 (0.0%)	*p* = 0.465
Previous CE stroke, *n* (%)	17 (19.1%)	7 (17.9%)	OR = 1.08 [0.41–2.86], *p* = 0.870
Time between strokes, years, median (IQR)	6 (1–27)	3 (1–21)	*p* = 0.682
AF known before previous CE stroke, *n* (%)	7 (41.2%)	3 (42.9%)	OR = 0.93 [0.16–5.54], *p* = 0.856

Abbreviations: OAC = oral anticoagulant, AIS = acute ischemic stroke, AF = atrial fibrillation, OR = (common) odds ratio, IQR = interquartile range, mRS = modified Rankin Scale, ICH = intracranial hemorrhage, CE = cardioembolic.

**Table 3:** Quality and type of anticoagulation of the 89 OAC AIS-AF patients.

Quality of anticoagulation, *n* (%)	
Under-anticoagulated	34 (38.2%)
Appropriately anticoagulated	48 (53.9%)
Over-anticoagulated	7 (7.9%)
Type of anticoagulation, *n* (%)	
DOAC	66 (74.2%)
Apixaban	43 (65.2%)
Edoxaban	6 (9.1%)
Rivaroxaban	9 (13.6%)
Dabigatran	8 (12.1%)
VKA	23 (25.8%)
Warfarin	10 (43.5%)
Acenocoumarol	13 (56.5%)

Abbreviations: OAC = oral anticoagulant, AIS = acute ischemic stroke, AF = atrial fibrillation, OR = (common) odds ratio, DOAC = direct oral anticoagulant, VKA = vitamin K antagonist.

**Table 4:** Demographic and clinical characteristics of the 89 OAC AIS-AF patients stratified by type of anticoagulation.

	DOAC (*n* = 66)	VKA (*n* = 23)	*p*-Value/OR [95% CI]
Demographic characteristics			
Age, years, median (IQR)	81 (46–95)	78 (50–91)	*p* = 0.184
Sex, male, *n* (%)	29 (43.9%)	10 (43.5%)	OR = 1.02 [0.42–2.51], *p* = 0.964
Medical history, *n* (%)			
Current smoking	4 (6.1%)	2 (8.7%)	OR = 0.68 [0.12–3.86], *p* = 0.657
Alcohol	30 (45.5%)	9 (39.1%)	OR = 1.29 [0.51–3.28], *p* = 0.588
Hypertension	61 (92.4%)	22 (95.7%)	OR = 0.61 [0.07–5.36], *p* = 0.653
Diabetes mellitus	27 (40.9%)	10 (43.5%)	OR = 0.90 [0.36–2.22], *p* = 0.820
Monitoring parameters, median (IQR)			
Weight, kg	77.4 (43–130)	-	-
Creatinine, µmol/L	91 (48–200)	-	-
GFR, mL/min/1.73 m^2^	55 (19–>90)	-	-
INR at admission	1.2 (0.9–2.4)	1.6 (1.0–6.9)	*p* = 0.345
pre-mRS score, median (IQR)	0 (0–4)	0 (0–4)	*p* = 0.629
pre-mRS score, *n* (%)			
0	37 (56.1%)	14 (60.9%)	OR = 0.84 [0.34–2.09], *p* = 0.693
1	11 (16.7%)	1 (4.4%)	OR = 4.04 [0.49–33.4], *p* = 0.187
2	9 (13.6%)	2 (8.7%)	OR = 1.66 [0.31–8.94], *p* = 0.558
>2	9 (13.6%)	6 (26.1%)	OR = 0.45 [0.13–1.39], *p* = 0.166
NIHSS score at admission, median (IQR)	6 (0–36)	7 (2–21)	*p* = 0.964

Abbreviations: OAC = oral anticoagulant, AIS = acute ischemic stroke, AF = atrial fibrillation, DOAC = direct oral anticoagulant, VKA = vitamin K antagonist, OR = (common) odds ratio, IQR = interquartile range, GFR = glomerular filtration rate, INR = international normalized ratio, pre-mRS = premorbidity modified Rankin Scale, NIHSS = National Institute of Health Stroke Scale.

**Table 5:** Outcomes of the 89 OAC AIS-AF patients stratified by type of anticoagulation.

	DOAC (*n* = 66)	VKA (*n* = 23)	*p*-Value/OR [95% CI]
Functional and safety outcome			
90-day mRS, median (IQR)	5 (0–6) *n* = 60	3 (0–6) *n* = 22	*p* = 0.379
Mortality at 90 days, *n* (%)	23 (34.9%)	7 (30.4%)	OR = 1.23 [0.47–3.24], *p* = 0.683
ICH, *n* (%)	3 (4.6%)	3 (13.0%)	OR = 0.32 [0.06–1.66], *p* = 0.176
Previous CE stroke, *n* (%)	13 (19.7%)	4 (17.4%)	OR = 1.17 [0.34–4.05], *p* = 0.800
Time between strokes, years, median (IQR)	6 (1–27)	5 (3–19)	*p* = 0.741
AF known before previous CE stroke, *n* (%)	4 (6.1%)	3 (13.0%)	OR = 0.43 [0.10–1.91], *p* = 0.267

Abbreviations: OAC = oral anticoagulant, AIS = acute ischemic stroke, AF = atrial fibrillation, DOAC = direct oral anticoagulant, VKA = vitamin K antagonist, OR = (common) odds ratio, IQR = interquartile range, mRS = modified Rankin Scale, ICH = intracranial hemorrhage, CE = cardioembolic.

**Table 6:** Demographic and clinical characteristics of the 89 OAC AIS-AF patients stratified by quality of anticoagulation.

	Under (*n* = 34)	Appro (*n* = 48)	Over (*n* = 7)	*p*-Value
Demographic characteristics				
Age, years, median (IQR)	82.5 (50–95)	79.5 (46–95)	80 (63–90)	*p* = 0.506
Sex, male, *n* (%)	14 (41.2%)	23 (47.9%)	2 (28.6%)	*p* = 0.590
Medical history, *n* (%)				
Current smoking	1 (2.9%)	5 (10.4%)	0 (0.0%)	*p* = 0.322
Alcohol	16 (47.1%)	21 (43.8%)	2 (28.6%)	*p* = 0.676
Hypertension	32 (94.1%)	44 (91.7%)	7 (100%)	*p* = 0.699
Diabetes mellitus	13 (38.2%)	19 (39.6%)	5 (71.4%)	*p* = 0.253
pre-mRS score, median (IQR)	0 (0–4)	0 (0–4)	0 (0–1)	*p* = 0.156
pre-mRS score, *n* (%)				
0	18 (52.9%)	27 (56.3%)	6 (85.7%)	*p* = 0.280
1	4 (11.8%)	7 (14.6%)	1 (14.3%)	*p* = 0.935
2	3 (8.8%)	8 (16.7%)	0 (0.0%)	*p* = 0.341
>2	9 (26.5%)	6 (12.5%)	0 (0.0%)	*p* = 0.118
NIHSS score at admission, median (IQR)	8 (0–36)	6 (0–24)	3 (1–11)	*p* = 0.239

Abbreviations: OAC = oral anticoagulant, AIS = acute ischemic stroke, AF = atrial fibrillation, Under = under-anticoagulated, Appro = appropriately anticoagulated, Over = over-anticoagulated, IQR = interquartile range, pre-mRS = premorbidity modified Rankin Scale, NIHSS = National Institute of Health Stroke Scale.

**Table 7:** Outcomes of the 89 OAC AIS-AF patients stratified by quality of anticoagulation.

	Under (*n* = 34)	Appro (*n* = 48)	Over (*n* = 7)	*p*-Value
Functional and safety outcome				
90-day mRS, median (IQR)	5 (0–6) *n* = 33	5 (0–6) *n* = 43	1.5 (0–6) *n* = 6	*p* = 0.349
Mortality at 90 days, *n* (%)	12 (35.3%)	17 (35.4%)	1 (14.3%)	*p* = 0.536
ICH, *n* (%)	3 (8.8%)	3 (6.3%)	0 (0.0%)	*p* = 0.692
Previous CE stroke, *n* (%)	5 (14.7%)	9 (18.8%)	3 (42.9%)	*p* = 0.231
Time between strokes, years, median (IQR)	6 (3–26)	5 (1–27)	6 (2–6)	*p* = 0.592
AF known before previous CE stroke, *n* (%)	4 (80.0%)	3 (33.3%)	0 (0.0%)	*p* = 0.067

Abbreviations: OAC = oral anticoagulant, AIS = acute ischemic stroke, AF = atrial fibrillation, Under = under-anticoagulated, Appro = appropriately anticoagulated, Over = over-anticoagulated, IQR = interquartile range, mRS = modified Rankin Scale, ICH = intracranial hemorrhage, CE = cardioembolic.

## Data Availability

The original contributions presented in the study are included in the article and further inquiries can be directed to the corresponding author.

## List of Abbreviations

OACsAF	Oral anticoagulants atrial fibrillation
TINL	Transzlációs Idegtudományi Nemzeti Laboratórium
CE	Cardioembolic
AIS	Acute ischemic stroke
NVAF	Non-valvular atrial fibrillation
DOACs	Direct oral anticoagulants
VKAs	Vitamin K antagonists
TIA	Transient ischemic attack
AHS	Acute hemorrhagic stroke
ECG	Electrocardiogram
GFR	Glomerular filtration rate
INR	International normalized ratio
pre-mRS	Premorbidity modified Rankin Scale
NIHSS	National Institute of Health Stroke Scale
mRS	Modified Rankin Scale
ICH	Intracranial hemorrhage
CT	Computer tomography
SPSS	Statistical Product and Service Solutions
SD	Standard deviation
IQR	Interquartile range
ANOVA	Analysis of variance
SHAP	Shapley Additive exPlanation
OR	Odds ratio
CI	Confidence interval
WARSS	Warfarin-Aspirin Recurrent Stroke Study
WASID	Warfarin-Aspirin Symptomatic Intracranial Disease
FDA	Food and Drug Administration
ESUS	Embolic stroke of undetermined source
RE-SPECT ESUS	Randomized Evaluation in Secondary Stroke Prevention Comparing the Thrombin Inhibitor Dabigatran Etexilate with Acetylsalicylic Acid (Aspirin) in Embolic Stroke of Undetermined Source
NAVIGATE ESUS	New Approach Rivaroxaban Inhibition in Embolic Stroke of Undetermined Source
AHRE	Atrial high-rate episodes
NOAH-AFNET 6	Non–Vitamin K Antagonist Oral Anticoagulants in Patients with Atrial High-Rate Episodes
ARTESiA	Apixaban for the Reduction of Thrombo-Embolism in Patients with Device-Detected Subclinical Atrial Fibrillation
BMI	Body mass index
PROFESS	Prevention Regimen for Effectively avoiding Second Strokes
SBP	Systolic blood pressure
LAAC	Left atrial appendage closure
RCT	Randomized control trial
ELPASE	Evaluation of Percutaneous Left Atrial Appendage Occlusion in Stroke Prevention on Top of Direct Oral Anticoagulant Therapy
LAA	Left atrial appendage
OCEANIC-AF	Oral Anticoagulant to Prevent Ischemic Stroke in Patients With Atrial Fibrillation (Asundexian) Trial
LIBREXIA-AF	LIBREXIA-Atrial Fibrillation: Evaluation of Milvexian in Stroke Prevention for Atrial Fibrillation Patients
LAAOS III	Left Atrial Appendage Occlusion Study III
